# A Review of Motion-Preserving Cervical Spinal Implants and Fusion Constructs

**DOI:** 10.3390/bioengineering13020228

**Published:** 2026-02-15

**Authors:** Isabella Merem, Rodrigo Vasquez, Jaden Wise, Elizabeth Beaulieu, Samip Patel, Maohua Lin, Gui Pires, Frank D. Vrionis

**Affiliations:** 1 College of Medicine, Florida Atlantic University, Boca Raton, FL 33431, USA; 2 Department of Biomedical Engineering, Florida Atlantic University, Boca Raton, FL 33431, USA; 3 SurGenTec LLC, Boca Raton, FL 33487, USA; 4 Department of Neurosurgery, Marcus Neuroscience Institute, Boca Raton Regional Hospital, Boca Raton, FL 33486, USA

**Keywords:** cervical spine biomechanics, motion-preserving spinal implants, spinal fusion, adjacent segment degeneration, range of motion, finite element analysis, load transmission

## Abstract

Spinal fusion remains a common surgical treatment for degenerative cervical spine pathology. By eliminating segmental motion, fusion alters spinal biomechanics and redistributes mechanical loads to adjacent levels. These changes contribute to adjacent segment degeneration (ASD). Motion-preserving spinal implants have been developed to address these limitations. Cervical disc arthroplasty (CDA) is the most widely used example. Such devices aim to maintain physiologic kinematics while preserving segmental stability. Their biomechanical behavior varies with implant design, material properties, and constraint characteristics. Previous research does not holistically compare fusion with motion-preserving treatments on the spine, resulting in an incomplete understanding of when motion-preserving devices should be considered in treatment over fusion constructs and which specific motion-preserving implants are most appropriate. This narrative review synthesizes experimental, computational, and clinical studies comparing rigid fusion constructs to motion-preserving technologies in the cervical spine. Emphasis is placed on segmental range of motion, load transmission, intradiscal pressure, facet joint forces, and adjacent-segment mechanics. By comparing effectiveness across motion-preserving treatments, alongside their effectiveness to fusion constructs, we found that CDA more closely preserves near-physiologic motion compared to fusion. Taken together, this review underscores the importance of biomechanics-informed implant design for guiding future innovation in spinal implant technologies.

## 1. Introduction

### 1.1. Clinical Role of Spinal Fusion

Anterior cervical discectomy and fusion (ACDF) remains one of the most commonly performed spinal procedures worldwide, with more than 100,000 cases performed annually in the United States alone [1]. Clinically, fusion is most frequently indicated for cervical radiculopathy or myelopathy when clinical and imaging findings support neural compression, and when symptoms are progressive and non-responsive to conservative care. The population-level burden of these conditions is substantial, as population-based data from Rochester, Minnesota, demonstrate an annual incidence of cervical radiculopathy of 107.3 per 100,000 in men and 63.5 per 100,000 in women, with peak incidence occurring between 50 and 54 years of age [2].

### 1.2. Biomechanical Consequences of Motion Elimination

From a biomechanical perspective, fusion disrupts normal segmental kinematics and redistributes motion and mechanical demand to adjacent spinal levels. Experimental and computational investigations have demonstrated increases in adjacent level range of motion, intradiscal pressure, and facet joint loading following fusion [3]. These changes alter load-sharing mechanisms within the spinal column and are widely implicated in the initiation and progression of adjacent segment degeneration (ASD). Accordingly, the annual incidence of symptomatic ASD following cervical fusion is estimated to range from 1.6% to 4.2% [4]. Furthermore, fusion constructs that terminate adjacent to, but do not include, the C5–C6 and/or C6–C7 levels are shown to be associated with an increased risk of developing ASD [4].

### 1.3. Rationale for Motion-Preserving Technologies

In contrast to rigid fusion constructs, motion-preserving technologies are spinal implants or surgical approaches designed to maintain or restore physiologic segmental motion at the treated level while providing sufficient stability [5]. Meta-analyses indicate that motion-preserving procedures are associated with significantly lower odds of radiographic adjacent segment degeneration (odds ratio [OR] 2.57 for fusion versus motion preservation) and reoperation due to adjacent segment pathology (OR 3.18) [6]. By preserving spinal kinematics and promoting more balanced load sharing across discs, facets, and ligaments, motion-preserving strategies aim to mitigate the biomechanical factors associated with fusion-related adjacent segment pathology.

### 1.4. Knowledge Gaps in Cervical Motion Preservation

Despite their shared goal of motion preservation, motion-preserving technologies differ greatly in implant design, constraint characteristics, and material properties, resulting in differences in biomechanical behavior. Prior reviews have largely focused on clinical outcomes or radiographic motion metrics, often aggregating motion-preserving devices as a single category. Prior studies have not compared the design-specific biomechanical consequences of various motion-preserving technologies in the cervical spine to fusion constructs at both index and adjacent spinal levels. As a result, it remains unclear when motion-preserving devices should be considered in treatment over fusion constructs, and which specific motion-preserving implants are most appropriate. Accordingly, this work presents a narrative, design-oriented biomechanical review comparing cervical spine biomechanics and motion-preserving technologies to clarify index-level and adjacent-level mechanical effects, inform implant selection, and guide future innovations in spine implant design.

## 2. Methods

### 2.1. Study Design

This study was conducted as a narrative literature review to synthesize existing experimental, computational, and clinical evidence describing the biomechanical effects of motion-preserving technologies compared with fusion constructs. A narrative approach was selected due to the substantial heterogeneity across study designs, loading protocols, and outcome normalization strategies in the cervical spine biomechanics literature, which precludes meaningful quantitative pooling or formal meta-analysis. Rather than estimating pooled effect sizes, the objective of this review was to integrate mechanistic insights and identify recurring biomechanical trends related to range of motion, load sharing, and adjacent segment mechanics across diverse study types. Where applicable, results were expressed as percent changes relative to intact or degenerated models as reported by the original authors, and differences in normalization methods were preserved and explicitly described.

### 2.2. Literature Identification and Scope

A comprehensive literature search was performed using PubMed, Google Scholar, OpenEvidence, institutional academic resources, and reference lists of relevant articles. The search included studies that were published from 1997 through 2025. The final search was performed on 7 February 2026. In PubMed, searches were performed using Boolean combinations of controlled vocabulary and keywords, including: (“cervical disc arthroplasty” OR “cervical disc replacement” OR “motion-preserving spine surgery”) AND (“biomechanics” OR “finite element” OR “cadaveric”) AND (“range of motion” OR “intradiscal pressure” OR “facet forces” OR “load sharing”). This search yielded 84 records, all of which were screened at the title/abstract level. Google Scholar searches were limited to the first 200 results per query, sorted by relevance, using similar keyword combinations. Duplicate records were removed manually prior to screening. Literature identification and selection were conducted by the first four authors through independent review followed by collective re-evaluation of selected articles. Differences in interpretation were resolved through discussion and consensus. No formal blinding to study authors, journals, or reported conclusions was applied, consistent with the narrative design of the review.

### 2.3. Evidence Selection and Narrative Synthesis

Studies were selected for inclusion based on their relevance to the biomechanical behavior of cervical spine implants, with emphasis on investigations reporting mechanistic outcomes such as range of motion, load sharing, and adjacent-segment mechanics. Rather than utilizing formal exclusion thresholds or scoring tools, studies were interpreted within the context of their methodological assumptions, model fidelity, and experimental constraints. Findings were synthesized narratively by comparing directional trends, relative magnitudes, and biomechanical behaviors across various implant designs. Studies focusing exclusively on surgical technique, clinical symptom scores, or radiographic outcomes without a biomechanical component were not a primary focus of this synthesis. Low-fidelity or weakly validated biomechanical and computational studies were not excluded or formally weighted. However, to address the potential influence of weakly validated computational studies on overall interpretation, we qualitatively compared the synthesized biomechanical trends with and without these studies. The removal of low-fidelity or highly idealized models did not materially alter the directional conclusions. Furthermore, greater interpretive emphasis was placed on findings supported by experimental validation or consistency across multiple studies.

For finite element analyses, model validity was assessed qualitatively at the level of the biomechanical variables reported rather than through formal quality scoring or statistical weighting. Emphasis was placed on whether the outcome variables analyzed were appropriate for the stated research question and whether those variables had established biomechanical relevance. In particular, studies were evaluated based on the criteria used by the original authors to justify the validity of the reported variables, including comparison with experimental or cadaveric data, consistency with known biomechanical behavior, and use of physiologic loading and boundary conditions. Findings from models lacking validation for the specific outcome variable or relying on highly idealized assumptions were interpreted cautiously but retained for completeness, as their inclusion did not change the overarching biomechanical patterns identified in this review.

### 2.4. Management of Bias

Given the narrative design of this review, formal risk-of-bias assessment and quantitative weighting of studies were not performed. Instead, potential bias and subjectivity were managed through several interpretive measures. First, identification of the literature was guided by predefined biomechanical outcome domains rather than study conclusions or implant claims. Second, evidence was synthesized across multiple study modalities, including cadaveric, computational, and translational clinical investigations, to reduce overreliance on any single experimental design. Third, biomechanical trends were emphasized only when observed consistently across multiple studies or methodological approaches. Isolated findings were contextualized accordingly and not treated as definitive.

### 2.5. Ordinal Biomechanical Scoring Framework 

Index-level changes in range of motion were classified using a unified ordinal scoring system based on normalized percentage change relative to the intact spine. Changes exceeding 20% were categorized as large increases (score = 2), increases ≤ 20% as moderate increases (score = 1), changes within ±5% as maintained (score = 0), decreases ≤ 15% as moderate decreases (score = −1), and decreases > 15% as large decreases (score = −2).

These thresholds are not intended as clinically validated cutoffs, nor do they represent definitive biomechanical failure or success criteria. Instead, they were chosen because the magnitudes correspond to functionally meaningful distinctions described in the literature on cervical biomechanics. For example, increases in motion on the order of 15–20% have been associated with measurable changes in segmental load sharing and facet joint contact patterns, which may influence pain generation and ASD. Conversely, changes within ±5% generally fall within the range of physiologic variability reported across cadaveric and computational studies and are unlikely to meaningfully alter motion perception or functional neck movement. Thus, although not formally validated, these domains were selected as pragmatic, directionally informative categories to support qualitative cross-study comparison while avoiding false precision in a heterogeneous literature.

## 3. Results

### 3.1. Fusion Biomechanics

#### 3.1.1. Effects of Segmental Motility and Stability

The designs of rigid fixation devices follow core biomechanical principles surrounding stability, implant stiffness, and load redistribution. The fixation procedure causes a loss of segmental range of motion, with experimental studies consistently demonstrating this relationship. Cadaveric studies report a decrease of over 75% in the degree of motion following fusion across flexion–extension, lateral bending, and axial rotation [7,8].

The degree of mobility restriction can be further impacted by the fusion technique, rigidity of the construct, and the number of levels fused. Higher-stiffness constructs, such as circumferential (360°) fixation, produce greater ROM restriction than less rigid models, such as anterior cervical plate fixation (ACPF). An in vitro study comparing these constructs found a 60% reduction in ROM following ACPF, which provided less stability, whereas the more stable 360° fusion resulted in a 79% ROM [9].

This stiffness-dependent relationship becomes more pronounced in multi-level fusions: as the number of fused levels increases, overall cervical spine ROM decreases progressively. A cadaveric study estimated that 1-level fusion constructs could still preserve between 84% and 96% of normal ROM across different planes of motion; however, these numbers decrease significantly with multi-level constructs, with a 5-level C2-C7 fusion preserving only 32% of normal lateral bending ROM [10].

#### 3.1.2. Effects on Adjacent Segments: Compensatory Hypermobility

The native intervertebral disc functions as a viscoelastic structure that absorbs and dissipates vibrational and impact loads through time-dependent deformation of the nucleus pulposus and annulus fibrosus, thereby attenuating forces transmitted across the functional spinal unit [11]. When this disc is removed and replaced with a rigid fusion construct, the natural load-sharing structure of the spine is altered, increasing the mechanical demands in adjacent intervertebral discs and facet joints [12]. As a consequence, adjacent spinal levels must take on a greater load in order to achieve the same range of motion [13,14].

These compensatory behaviors can manifest as hypermobility, elevated disc stress, altered facet loading, and increased ligament forces, all of which contribute to complications such as ASD. In fact, as many as 92% of patients undergo degenerative changes at adjacent segments, with 1 in 4 having symptomatic disease in the first 10 years after fusion [14].

The loss of motion at fused segments results in compensatory hypermobility at adjacent unfused levels. Single-level fusion increases ROM at adjacent segments by varying degrees depending on the motion plane and specific level fused, with more significant compensation at the levels immediately adjacent to fusion [15]. Similarly, FEA studies have found a significant increase in adjacent ROM following both one- and two-level cervical fusions [16]. Across multiple models, a consistent trend has been observed between ROM loss at the fused segment and hypermobility at adjacent levels.

#### 3.1.3. Additional Effects on Adjacent Segments, Load Redistribution, and ASD

Adjacent-segment hypermobility after fusion directly alters the spine’s natural mechanical demands and shifts additional loads to neighboring levels. Studies of cervical fusions consistently report a significant rise in adjacent-segment intradiscal pressure (IDP). For example, in a study by Eck et al. [3], IDP at segments adjacent to a cervical fusion increased by up to 73.2% at the superior adjacent level and by 45.3% at the inferior level during flexion [3]. The greater pressure increases at the superior adjacent level correspond with greater segmental motion at that level. This superior-level hypermobility may predispose the segment to kyphotic alignment changes, potentially accelerating degenerative processes [3].

Rises in IDP are also associated with loss in disc height, increased strain, and accelerated degeneration [3,17,18,19]. Fusion also alters facet joint forces, shear loads, and tissue stress in adjacent segments. More rigid fixation devices appear to amplify these effects: higher stiffness not only produces greater ROM loss, but also generates greater changes in IDP, stress, and facet joint contact forces [17,20]. Together, these mechanical alterations contribute to the development and progression of ASD [18,20].

#### 3.1.4. Materials and Designs of Fusion Devices

The biomechanical outcomes described above are shaped by the materials and designs of the devices. Rigid materials (e.g., titanium cages and rods) increase segmental stiffness, which can exacerbate the compensatory mechanisms and biomechanical changes at adjacent segments. Non-porous titanium (Ti) interbody fusion devices also show a higher risk of subsidence [21]. Nevertheless, their rigidity provides greater stability and promotes bony fusion and healing [22]. In vitro experiments have shown that human osteoblasts can grow through titanium foam, while expressing osteogenic genes, and Ti-grown osteoblasts produce higher levels of angiogenic and osteoprotegerin compared to polyetheretherketone (PEEK) cultures [23,24].

Conversely, less rigid materials, such as PEEK, provide better load redistribution and more modest increases in shear stress and IDP, while also having a lower risk of subsidence [21]. However, PEEK’s chemical inertness makes it less biocompatible, and its lower segmental stability can further impair bone integration and fusion rates [22,25].

Fortunately, surface modification strategies can mitigate these limitations by altering the properties of these materials. Devices made from different titanium alloys can exhibit altered porosity and rigidity, and plasma treatment can enhance PEEK’s biocompatibility, all of which can improve osteointegration [22,24,25].

Overall, fusion device design and selection require careful consideration and balancing of the various trade-offs in relation to stability, bone healing efficiency, material stiffness, and the downstream biomechanical consequences that can lead to further complications. These principles remain true for other constructs, such as structural allografts, biodegradable rods and cages, and other emerging fusion technologies [26,27].

### 3.2. Motion-Preserving Technologies

#### 3.2.1. Biomechanical Rationale for Cervical Disc Arthroplasty

Motion-preserving technologies in the cervical spine have emerged as alternatives to fusion procedures such as ACDF to address the recognized biomechanical drawbacks of rigid constructs. CDA, the most extensively studied motion-preserving intervention, involves implanting an artificial disc after the removal of the original disc [28]. It is designed to maintain segmental range of motion and promote more natural load sharing across the spine’s vertebrae.

**Figure 1:**
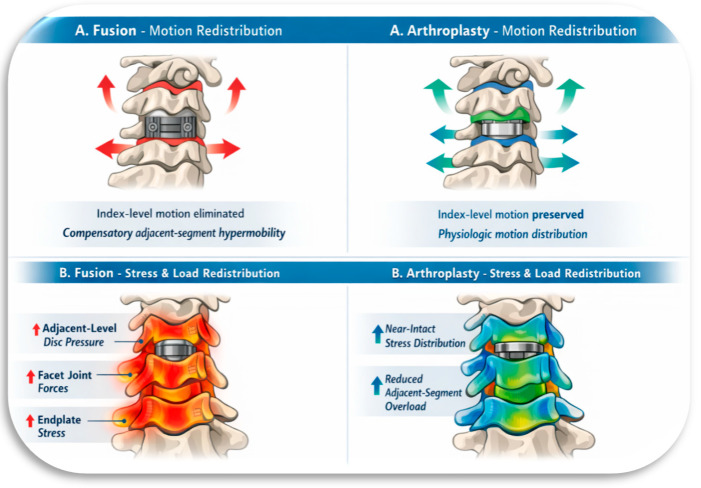
Comparison of cervical spinal fusion and motion-preserving arthroplasty.

Cadaveric studies have demonstrated distinct biomechanical differences between cervical fusion and motion-preserving technologies. In a direct comparison of intact, fused, and prosthetic cervical motion segments, Dahl et al. [14] demonstrated that fusion constructs exhibit significantly increased dynamic stiffness relative to intact segments, whereas prosthetic segments displayed dynamic stiffness comparable to intact discs, indicating preservation of physiologic motion behavior. Complementary stress-based analyses further demonstrated that fusion increased intradiscal pressure and stress concentrations at adjacent levels by approximately 50%, whereas CDA redistributed applied loads through the implant, resulting in reduced abnormal stress transfer to neighboring segments [12]. The fundamental biomechanical distinction between fusion and motion-preserving arthroplasty is illustrated schematically in Figure 1.

**Figure 2:**
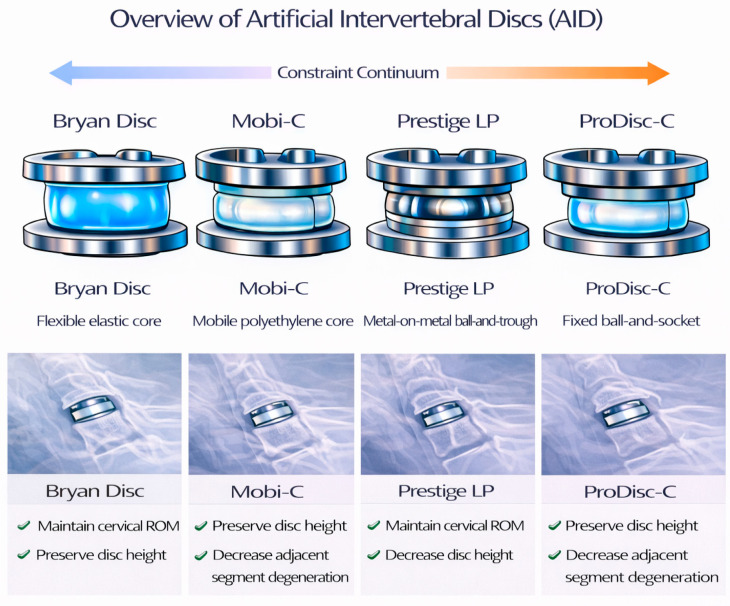
Design spectrum of cervical disc arthroplasty devices.

Although cervical disc arthroplasty is broadly categorized as a motion-preserving intervention, implant designs vary substancially in their constraint mechanisms and articulation strategies. As illustrated in Figure 2, these devices span a continuum of constraint rather than representing a single biomechanical solution, which contributes to variable outcomes.

#### 3.2.2. Effects on Segmental and Adjacent-Level Kinematics

**Figure 3:**
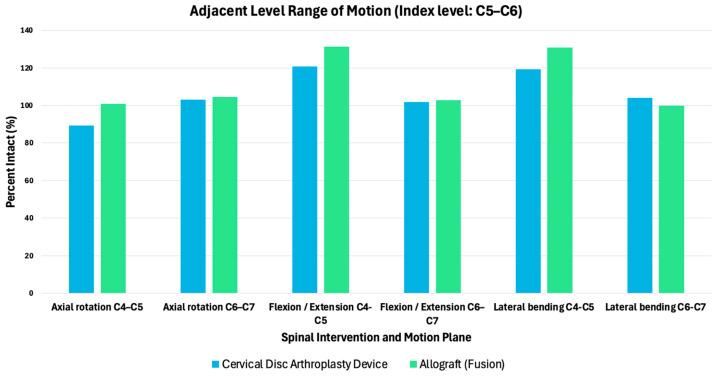
Adjacent-level range of motion following CDA and fusion, normalized to the intact condition (index level: C5x–C6). Percent motion at adjacent segments (C4–C5 and C6–C7) is shown for axial rotation, flexion–extension, and lateral bending based on cadaveric testing [29]. Values are reported as a percentage of intact motion. Data adapted from Dmitriev et al. [25].

Biomechanical distinctions comparing the motion distribution of fusion against motion-preserving constructs at index and adjacent spinal levels are further illustrated in Figure 3. As shown in Figure 3, cervical fusion resulted in increased adjacent-level range of motion across axial rotation, flexion–extension, and lateral bending when normalized to the intact condition, with superior adjacent levels demonstrating increases exceeding 130% of intact motion in certain loading modes [29]. In contrast, CDA maintained adjacent-level motion closer to intact values across all planes.

To synthesize these findings, Table 1 summarizes reported changes in index level and adjacent-level kinematics following motion-preserving techniques, including arthroplasty, and fusion across several studies. Despite variability in implant design and modeling approach, fusion constructs consistently demonstrate reduced index level motion with compensatory increases in adjacent-segment motion and loading, whereas motion-preserving devices more closely preserve near-physiologic kinematics and mitigates adjacent-level hypermobility.

**Figure 4:**
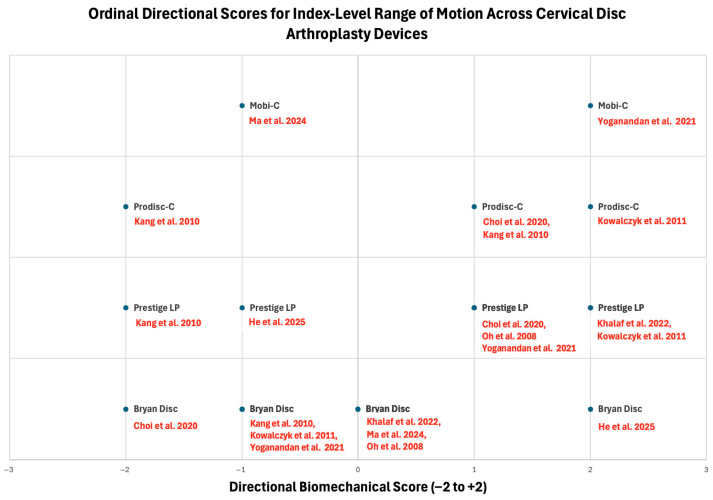
Ordinal directional changes in index level range of motion across cervical disc arthroplasty devices [32,33,34,35,36,37,38,39]. Scores reflect direction and relative magnitude of change (−2 to +2) rather than absolute kinematic values. Individual data points are labeled by prosthesis type and source study.

The direction and magnitude of preserved mobility of cervical disc arthroplasty varies substantially across prosthesis designs. Different implants result in varied effects on segmental kinematics that are difficult to compare directly using absolute range of motion values alone. To facilitate cross-study comparison, reported index level range of motion changes were synthesized using an ordinal directional scoring framework that captures both the direction and relative magnitude of biomechanical change (Figure 4). Less constrained designs, including Mobi-C and ProDisc-C, more frequently exhibit increased segmental range of motion relative to reference conditions, consistent with greater freedom of articulation and less resistance to sagittal and coupled plane movement. In contrast, devices such as Bryan and Prestige LP tend to maintain or modestly constrain index level mobility across studies, with outcomes influenced by study conditions and loading paradigms [32,33,34,35,36,37,38,39]. These kinematic differences provide essential context for interpreting how differences in motion-preserving implants redistribute loads to the facet joints, lateral cervical joints, and adjacent intervertebral discs.

#### 3.2.3. Load Redistribution and Mechanistic Insights from Finite Element Analysis

Although cadaveric and clinical studies consistently demonstrate differences in motion redistribution between fusion constructs and motion-preserving implants, they offer limited insight into the specific biomechanical mechanisms driving these observations. Parameters such as endplate stress, facet joint loading, and adjacent-level strain are difficult to isolate or quantify using in vivo or cadaveric models alone. FEA provides a complementary approach by enabling controlled evaluation of spinal biomechanics under defined loading conditions, permitting systematic comparison of fusion and motion-preserving constructs beyond what is achievable through experimental or clinical studies.

FEA quantifies how fusion and motion-preserving implants redistribute motion and mechanical loads across treated and adjacent spinal segments. Rigid fusion constructs using pedicle screws and stainless steel or titanium rods nearly eliminate motion at the index level, with modeled reductions in segmental range of motion of approximately 44–54% across physiologic loading modes. This immobilization results in redistributed mechanical demands at adjacent levels, including increases in intradiscal pressure, annulus fibrosus stress exceeding 25%, elevated facet joint forces, and adjacent level ROM increases of approximately 15–18%. These are changes that have been implicated as contributors to ASD [20,40,41].

In contrast, finite element models of total disc replacement (TDR) demonstrate preservation of near-normal motion and stress distribution at the surgical level, with adjacent segment mechanics remaining within approximately 7% of intact spine values [40,41]. Artificial disc replacement is associated with increased motion at the treated level, with approximately 50% greater motion during extension and 20% greater motion during flexion compared with the intact spine [41]. This increase occurs without the adjacent-level stress amplification observed with fusion constructs. However, certain disc designs may generate excessive ligament tension (>500 N) and elevated facet joint pressures (>3 MPa), indicating that motion preservation is dependent on implant design and constraint characteristics [29].

Across finite element studies, biomechanical differences between fusion and disc replacement are most pronounced during flexion, followed by lateral bending, extension, and axial rotation [35].

#### 3.2.4. Implant-Dependent Global Cervical Motion Patterns

In contrast to cervical fusion, which eliminates the native disc’s capacity to attenuate vibrational and impact loads, CDA partially restores the shock-absorbing function of the functional spinal unit. Nevertheless, load dissipation remains highly dependent on implant design, and current prostheses do not fully reproduce the viscoelastic behavior of the native intervertebral disc [12].

**Figure 5:**
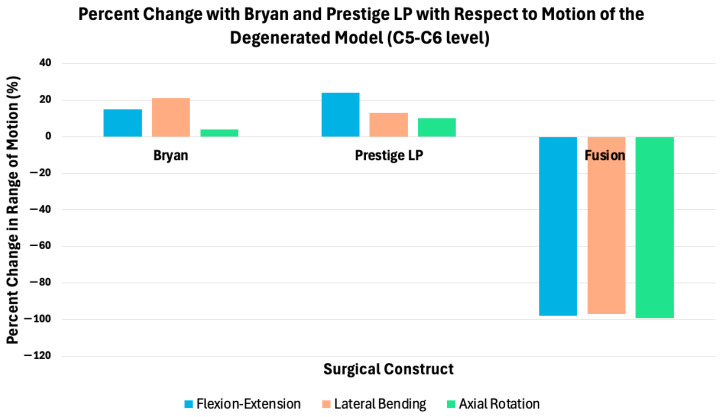
Percent change in segmental range of motion at index level (C5–C6) relative to a degenerated cervical spine model following disc arthroplasty and fusion. Bar plots depict percent changes in flexion–extension, lateral bending, and axial rotation at the treated C5–C6 level for two cervical disc arthroplasty devices (Bryan and Prestige LP) and fusion, normalized to the degenerated model.

Using a validated three-dimensional finite element model of the cervical spine, Gandhi et al. [31] compared intact, fused, and arthroplasty constructs employing Bryan and Prestige LP discs. Specifically, total disc arthroplasty using the Bryan device was associated with modest increases in flexion–extension and lateral bending, with minimal changes in axial rotation, whereas implantation of the Prestige LP disc demonstrated comparatively greater increases in motion across these planes [31]. In contrast, fusion reduced motion at the treated spinal level and increased motion at adjacent cervical segments (Figure 5).

**Figure 6:**
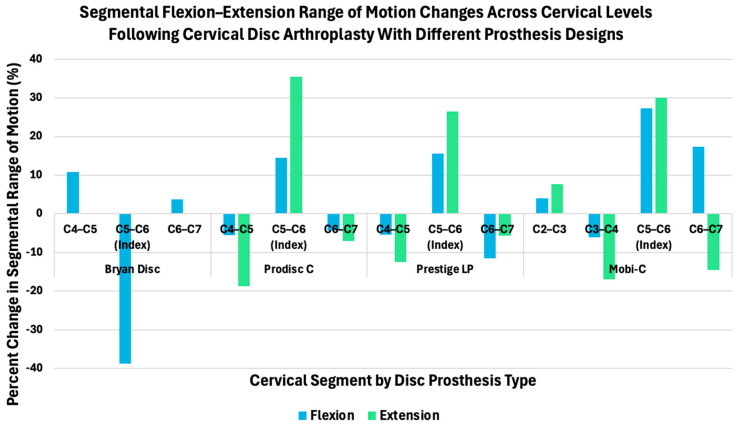
Percent change in segmental range of motion during flexion and extension across cervical levels following CDA with different prosthesis designs. Segmental motion changes at the index (C5–C6) and adjacent levels are shown for Bryan, ProDisc-C, and Prestige LP implants based on finite element analyses reported by Choi et al. (2020) [32], which evaluated motion across C4–C7 (C4–C5, C5–C6, and C6–C7). Corresponding data for the Mobi-C implant are adapted from Jiang et al. (2023) [42], which assessed segmental kinematics across a broader cervical range (C2–C7), including C2–C3, C3–C4, C5–C6, and C6–C7. All values are reported as percent change in segmental range of motion relative to the respective intact or baseline model used in each study.

Hybrid constructs combining fusion and disc replacement further illustrate how implant design influences segmental kinematics across cervical levels. Finite element analyses by Choi et al. [32] demonstrated that different prosthesis designs produce distinct patterns of flexion–extension redistribution at the index (C5–C6) and adjacent levels (C4–C5 and C6–C7). Specifically, Bryan disc arthroplasty resulted in a reduction in index level flexion with relatively small changes in extension, reflecting a more constrained motion profile [32]. In contrast, ProDisc-C and Prestige LP devices showed substantial increases in both flexion and extension at the treated level, as well as reduced motion at adjacent segments (Figure 6) [32]. When complementary Mobi-C data adapted from Jiang et al. [42] is considered, these effects extend across a broader cervical range (C2–C7), highlighting how prosthesis constraint, design, and mobility characteristics influence both local and global motion distribution following motion-preserving cervical surgery [32,42].

**Figure 7:**
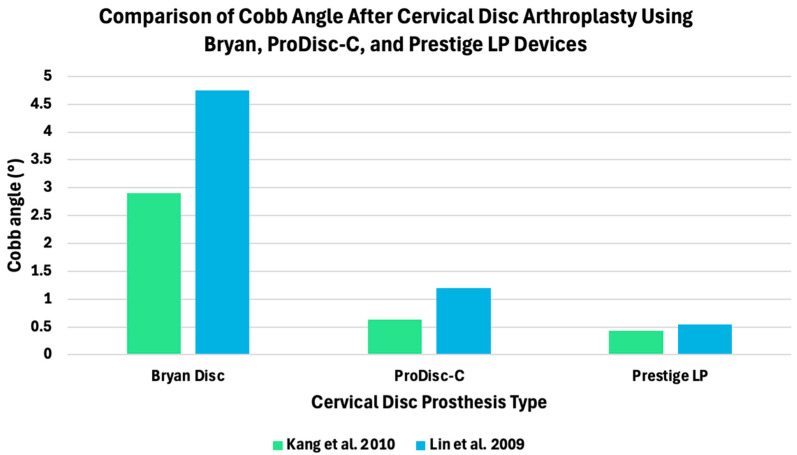
Segmental range of motion measured at the index level following cervical disc arthroplasty with different prosthesis designs. Values represent Cobb angle-based range of motion under flexion–extension loading, as reported by Kang et al. (2010) [34] and Lin et al. (2009) [43].

Differences in segmental motion are also reflected in absolute angular measurements of spinal motion. Cobb angle-based assessments reported by Kang et al [34] and Lin et al [43] quantify the angular range of motion between adjacent vertebral endplates during flexion–extension, providing a clinically interpretable measure of segmental mobility at the index level. Using this metric, substantial variation in flexion–extension range of motion is observed across commonly used cervical disc prostheses [34,43]. The Bryan disc exhibited the largest segmental angular range of motion, whereas ProDisc-C and Prestige LP exhibited progressively smaller Cobb angles, consistent with increasing implant constraint (Figure 7).

When motion is considered at the level of the entire cervical spine rather than the index segment alone, these differences translate into measurable changes in global cervical kinematics. In a systematic clinical and radiographic analysis, Anderson et al. [44] creported that patients undergoing cervical arthroplasty demonstrated significantly greater global C2–C7 range of motion compared with those treated with ACDF, with combined estimates favoring arthroplasty by approximately 3–5° at short-term follow-up [44]. This preservation of motion persisted with long-term follow-up. Additionally, arthroplasty was associated with more lordotic sagittal alignment at adjacent segments, whereas fusion tended to concentrate motion across fewer remaining mobile levels [44]. In another study by Auerbach et al. [45], patients who underwent arthroplasty demonstrated a statistically greater improvement in total cervical ROM (+5.9°) compared to those who underwent an ACDF (−0.8°) at 2 year follow-up [45]. These findings suggest that while adjacent segment kinematics may be similar between procedures in the short term, cervical arthroplasty provides more preservation of global cervical motion and alignment, factors that may influence long-term load distribution and biomechanical stress across the cervical spine.

These biomechanical and kinematic findings indicate that motion-preserving cervical implants offer meaningful advantages over fusion, but that the extent of motion preservation is highly dependent on procedure type, level treated, and implant design and positioning, portraying the importance of device-specific evaluation when interpreting long-term clinical outcomes for patients.

#### 3.2.5. Long-Term Data

**Figure 8:**
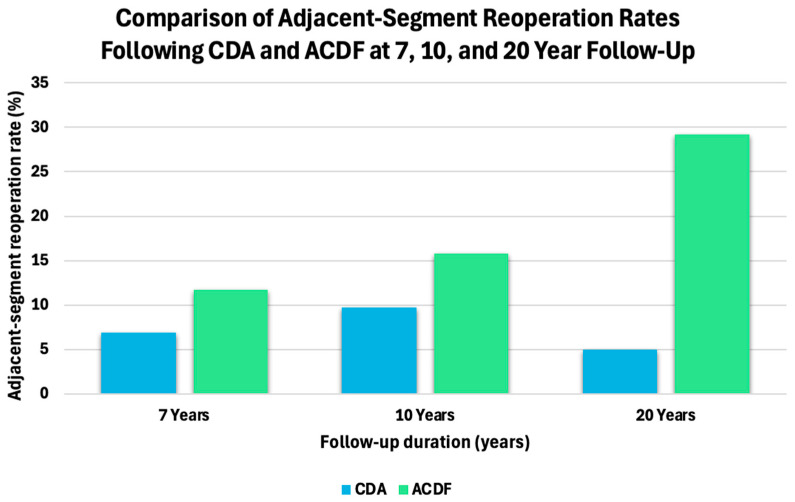
Adjacent-segment reoperation rates following CDA and ACDF at 7-, 10-, and 20-year follow-up. Bar plots summarize reported adjacent-segment reoperation rates across long-term randomized trials and registry-based studies. Values are compiled from studies with differing designs, patient populations, and follow-up durations and are not derived from a single cohort [47,48].

The biomechanical advantages of motion-preservating devices are becoming increasingly supported by long-term clinical evidence. A large 2025 meta-analysis by Yakdan et al. [46] including 25 randomized controlled trials and 4530 patients demonstrated significantly lower overall and adjacent-segment reoperation rates following CDA compared with ACDF, despite longer operative times [46]. As summarized in Figure 8, reported adjacent-segment reoperation rates are consistently lower following CDA than ACDF at 7-, 10-, and 20-year follow-up intervals across long-term clinical studies. Long-term randomized data from Bryan and Prestige disc trials further support this trend, with combined 10-year follow-up data demonstrating adjacent-segment surgery rates of 6.9% after CDA versus 11.7% after ACDF [47]. At extended follow-up, a 20-year randomized trial of the Bryan disc reported reoperation rates of 10% for CDA compared with 41.7% for ACDF [48]. However, registry-based studies with mid-term follow-up have reported no significant difference in adjacent-segment reoperation risk between single-level CDA and ACDF, emphasizing the influence of patient selection and follow-up duration on observed clinical outcomes [49].

Radiographic ASD is frequently observed following both fusion and arthroplasty and does not perfectly translate to clinical symptoms. Long-term imaging studies show lower radiographic ASD severity at adjacent levels following CDA compared to ACDF, yet symptomatic ASD requiring reoperation occurs in only a minority of patients [50,51]. Heterotopic ossification (HO) increases progressively with time of follow-up, with motion-restricting HO affecting approximately 30–42% of patients at 10 years follow-up [52]. Taken together, these findings suggest that while CDA does not fully prevent degenerative progression, its preservation of more physiologic motion patterns may contribute to lower adjacent-segment reoperation rates over long-term follow-up.

#### 3.2.6. Limitations in Long-Term Data

Interpretation of long-term clinical outcomes comparing motion-preserving devices and fusion constructs is limited by heterogeneity in study design, patient selection, and duration of follow-up across existing clinical trials and registries. For instance, long-term data coming from older generation devices are subject to survivorship bias, which may underestimate late complications and reoperation risk. Furthermore, differences in ASD definitions and radiographic measurements make comparisons between studies difficult. Long-term studies comparing how motion-preserving device-specific materials influence biomechanical effects in comparison to fusion constructs are needed to more accurately assess the durability of motion-preserving devices as a whole. Additional long-term studies should also be conducted to determine how changes in segmental and global cervical kinematics mediate the relationship between ASD, HO progression, and other clinically meaningful outcomes.

### 3.3. Case Studies

To contextualize the biomechanical principles outlined above, individual case studies provide insight into how fusion and motion-preserving constructs alter segmental and adjacent-level behavior under controlled experimental conditions [31,53].

#### 3.3.1. Cadaveric Studies

Cadaveric spines preserve native anatomy, bone quality, ligamentous constraints, and joint mechanics, allowing investigators to evaluate implants under conditions that closely approximate in vivo behavior [54]. This preservation of physiologic loading makes cadaveric testing well-suited for assessing differences in segmental and adjacent-level range of motion following arthroplasty and arthrodesis. In an 18-specimen cadaver case study at the C6–7 level, Chang et al. [17] demonstrated that artificial discs increased mobility at the treated level while modestly reducing motion at adjacent levels, with IDP changes remaining within 10% of the intact spine. In contrast, ACDF significantly reduced index-level motion and induced compensatory biomechanical changes at adjacent levels, including an IDP increase of about 33.9–46.5% at the superior level, and increases in facet forces of approximately 28% and 24% at superior and inferior levels, respectively, relative to intact conditions [17]. Similar motion-preserving and adjacent-level offloading patterns have been reported in additional cadaveric studies across cervical regions, further supporting the concept that arthroplasty better maintains native kinematics than arthrodesis [7,30,55]. The motion patterns observed across these cadaveric models are consistent with previously described associations between adjacent-segment mobility and increased intradiscal pressure, facet loading, and ligamentous strain.

#### 3.3.2. Clinical Outcomes

Clinical outcome data broadly corroborate the biomechanical advantages observed in cadaveric testing. Designs with greater translational freedom reduce intrinsic constraint, which may enhance segmental stability while simultaneously increasing facet joint loading under certain motion patterns [56]. In contrast, mobile-core polyethylene systems such as Mobi-C permit controlled translation of the core relative to the endplates, enabling adaptive shifts in the instantaneous center of rotation that may better accommodate physiologic motion, but reduce intrinsic constraint. This is reflected in greater increases in segmental ROM across all planes in mobile-core compared to fixed-core designs, notably during axial rotation (105% vs. 45%, respectively), and is also accompanied by substantial load redistribution, including facet force increase of about 210% in both design types [57].

Across these designs, differences in constraint mechanism and material composition produce distinct kinematic behavior, center of rotation characteristics, and load transfer profiles that influence facet joint forces, intradiscal pressure, and adjacent-level motion. Consequently, biomechanical outcomes attributed to cervical disc arthroplasty often reflect device-specific design properties rather than a uniform effect of motion preservation itself. These design-dependent differences in constraint mechanism, motion behavior, and load transfer across commonly used CDA systems are summarized in Table 2.

#### 3.3.3. Finite Element Analysis Complexity

Computational complexity further limits direct comparison between fusion and arthroplasty. Traditional finite element models often rely on simplified material properties, static loading assumptions, and idealized boundary conditions, with soft-tissue attachments typically estimated from general anatomical data rather than patient-specific measurements. As a result, anatomical variations, such as spinal curvature and disc height, might not be captured in generalized models, which inadequately represent the dynamic, multi-directional behavior of motion-preserving implants. For example, under physiological loading, a kyphotic curvature at C5–C6 was found to have reduced disc motion by 30% under flexion and 23% under extension, while increased or decreased disc height changed extension responses by +8.3% and −12%, respectively [58]. Accurately modeling articulating surfaces, viscoelastic cores, wear mechanisms, and time-dependent changes in implant–bone interaction remains computationally demanding. Although advanced and AI-assisted modeling approaches offer improved efficiency and scalability, they remain inconsistently validated across devices and loading conditions, restricting their current utility for standardized comparative analysis [59,60,61].

### 3.4. Modeling and Regulatory Limitations

#### Regulatory Processes

Finally, regulatory and adoption constraints remain an important barrier to direct comparison between fusion and motion-preserving arthroplasty. Motion-preserving implants are evaluated through device-specific regulatory approval pathways that require long-term follow-up, which has limited the number of large, randomized comparative trials available for analysis [48,62]. In addition, differences in approval timing and indications across regions have resulted in uneven clinical adoption, with some arthroplasty devices widely used internationally while remaining restricted or investigational elsewhere [63]. These factors contribute to variability in reported outcomes and reduce the strength of pooled comparisons between fusion and motion-preserving strategies.

These limitations show that differences between fusion and motion-preserving arthroplasty are influenced not only by biomechanics, but also by gaps in long-term data, device-specific design differences, modeling constraints, and regulatory factors. Until these issues are better addressed, it remains difficult to define which patients derive durable benefit from motion preservation compared with fusion.

## 4. Future Directions

As research in spinal implants and spine surgery progresses, motion-preserving spinal implants represent a huge step forward from traditional fusion techniques and standardized implant models [64]. Most standardized implants imperfectly represent individual patients disc space anatomy; new personalized approaches would leverage advanced imaging and FEA to create unique implants according to each patient’s spinal anatomy and loading conditions [65,66]. This integration helps to address the common aforementioned clinical challenges which persistently compromise outcomes in spinal fusion and implant surgery. Furthermore, the merging of FEA and other deep-learning technologies have made it feasible to produce these devices in a clinically relevant timeline, shifting personalized implants from being an experimental innovation to a practical tool [66,67,68,69].

### 4.1. Proposed Workflow

**Figure 9:**
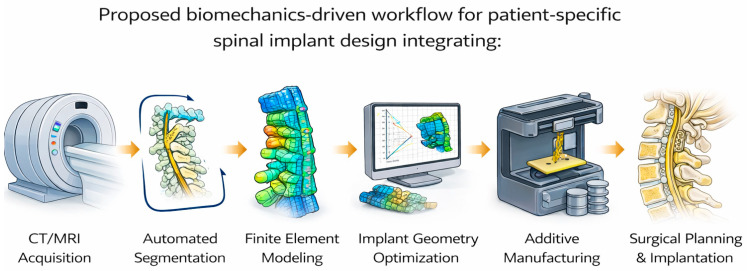
Biomechanics-driven workflow for patient-specific spinal implant design.

An overview of the proposed biomechanics workflow for patient-specific spinal implant design is shown in Figure 9.

### 4.2. Image Acquisition and Segmentation

Image acquisition and segmentation are the critical foundations of biomechanics-driven spinal implant workflows. This allows clinicians to transform raw imaging data into anatomical data which drives computational modeling and implant design. CT imaging data is the standard imaging modality for personalized implants because if its unique ability for bone visualization and compatibility with FEA [67]. MRI-based workflows are more variable, however, but necessary when there is a greater need for soft-tissue visualization. These CT or MRI images can then be processed using deep learning-based technologies [70]. This includes multiclass segmentation of cropped 3D CT images using neural networks such as DenseVNet. This process is able to identify and differentiate the various components of the vertebral column from one another; including but not limited to cortical bone, cancellous bone, cartilage, and IVDs including the nucleus pulposus and fibrous annulus [71]. In lumbar spine models, these automated processes are able to reduce processing time from days to hours and minimize user variability, which is likely to be comparable to cervical models [67].

### 4.3. Biomechanical Modeling and Simulation

After segmentation, anatomical structures should be converted into computational meshes for FEA. Nispel et al. [72] developed an automated pipeline through which surface meshes underwent Laplacian smoothing and adaptive decimation. This was done to ensure seamless contact surfaces between vertebrae and discs while preventing computational artifacts at tissue interfaces [72]. Ligament placement must also be considered in creating biomechanical models [72]. Current innovations include coordinate-based frameworks employing spherical coordinate segmentation to automate ligament placement and orientation. After a complete anatomical representation of the spine is achieved, simulations apply physiological loading scenarios to the model [73]. This includes pure moment loading such as flexion, extension, lateral bending, and axial rotation [74]. Pure compression simulating body weight and muscle forces is analyzed. Combined load is also reviewed to evaluate how the model acts under the stress of daily activities [73]. Finally, these models are then validated against experimental data. This is done by comparing predicted outcomes against published experimental findings from cadaveric studies. Metrics for validation include range of motion, facet contraction forces for all loading directions, intradiscal pressure under compression, and combined loading.

### 4.4. Implant Design Optimization

Following biomechanical simulation, patient-specific finite element models can be used to evaluate and refine implant geometry to improve load sharing and reduce stress concentrations at the bone–implant interface. Prior biomechanical studies of spinal implants have demonstrated that implant geometry and stiffness strongly influence endplate stress, subsidence risk, and segmental range of motion, supporting the use of implant geometry optimization approaches as opposed to standardized designs [7,13].

Customizable implant design strategies allow implant dimensions, lordotic angles, and contact surfaces to be adjusted based on patient-specific endplate morphology and bone quality, which may mitigate stress shielding and excessive forces that contribute to subsidence and adjacent segment degeneration [12,28]. These approaches are particularly relevant in osteoporotic bone, where mismatches between implant stiffness and vertebral strength can compromise fixation.

Implant material properties also represent a critical design variable influencing axial load transfer and endplate stress. Material-level biomechanical testing has demonstrated that more compliant, energy-dissipating cervical disc materials exhibit lower dynamic stiffness and greater viscous damping compared with fusion constructs, resulting in axial shock absorption closer to that of the native disc and reduced stress transmission to the vertebrae (Table 3) [14]. These characteristics provide a mechanistic basis for improved load sharing and reduced subsidence risk.

Recent advances in computational biomechanics suggest that surrogate modeling approaches may further enhance this workflow by reducing the computational burden associated with repeated finite element analyses. For instance, machine learning assisted frameworks have been proposed to approximate biomechanical outputs based on prior simulation data [75]. These approaches have been shown to accurately approximate vertebral stress distributions while reducing simulation times from hours to seconds, thus improving the feasibility of patient assessments in the clinic [75]. Although these methods are still emerging in spine applications, they represent a promising direction for translating patient-specific optimization into clinically realistic timelines [75].

### 4.5. Manufacturing and Validation

The next step in the workflow of biomechanics-driven personalized spinal implants involves additive manufacturing and computational model validation. Additive manufacturing allows manufacturers and physicians to create geometrically complex patient-specific designs [76]. The most common technique is selective laser smelting. This builds devices layer by layer from titanium powder [76]. Manufacturing must accommodate the unique anatomical features of each patient. This may be achieved through investigational approaches such as topology optimization, which has been shown to significantly reduce subsidence risk in certain studies [77]. Prototypes of implants must also undergo mechanical testing to validate the findings of FEA models [76]. Computation models are measured against ASME standards, which includes verification of FEA predictions against mechanical testing data and validation of subsidence risk predictions [76]. Clinical outcome validation can also be done post operatively through reporting radiographic, biomechanical, and patient reported outcomes. Key validation benchmarks include fusion rates, positioning accuracy, subsidence occurrence, and functional outcomes.

### 4.6. Surgical Implementation

Finally, surgical implementation of personalized spinal implants leverages pre-operative planning, patient specific instrumentation, and pre-selected implant kits. Pre-operative planning and kit preparation involves using patient CT data to create a 3D printed biomodel of the patient’s spine for planning and patient education [78]. This biomodel then serves as an intraoperative reference for the surgeons. This enables accurate placement of instruments and subsequent screw and implant placement accuracy [78]. Thayaparan et al. [78] employed pre-surgical planning and 3D-printed bio models to prepare patient specific kits for assistance in spinal surgery. They found that pedicle screw placement was 97.8% accurate on post operative CTs and that pre-selected kit implants exactly matched intraoperative measurements [78].

In summary, the proposed workflow integrates imaging, biomechanical modeling, implant optimization, manufacturing validation, and surgical planning into a framework for personalized spinal implant development. Deep learning-based implant processing and FEA simulations enable anatomical accuracy and biomechanically informed decision making. This ultimately reduces subsidence risk, optimizes load sharing, and enhances surgical precision. By incorporating data driven optimization strategies, efficient customization is supported while clinical restraints of time are addressed. Collectively, this workflow moves away from the traditional one size fits all implant approach and moves towards truly patient-specific solutions with the potential for improved mechanical performance, surgical outcomes, and long-term patient function in spinal implants.

## 5. Conclusions

Spinal implant technology is undergoing a significant transformation, evolving from rigid stabilization strategies towards more biomechanically informed, motion-preserving strategies. As synthesized throughout this review, while traditional spinal fusion techniques remain effective for restoring spinal stability, they do so at the cost of reducing spinal kinematics and load transmission, resulting in markedly decreased index level motion and compensatory increases in motion, stress, and intradiscal pressure at adjacent segments.

In contrast, motion-preserving technologies, including cervical disc arthroplasty, dynamic stabilization systems, and emerging hybrid constructs, have been shown across cadaveric testing, finite element simulations, and clinical studies to maintain improved physiologic segmental motion and more balanced load sharing. The preservation of native or near-native kinematics limits compensatory hypermobility at adjacent levels and reduces abnormal stress transfer to discs, facet joints, and supporting ligaments. While these biomechanical advantages do not yet ensure long-term prevention of adjacent segment pathology, they represent a meaningful step toward addressing the root mechanical drivers of degeneration rather than merely stabilizing symptomatic levels.

Notably, biomechanical performance is not determined solely by whether an implant preserves motion or enforces rigidity, but by nuanced interactions among implant design, material properties, constraint mechanisms, and patient-specific anatomy. Variability among motion-preserving devices, such as unconstrained versus semi-constrained articulation, produces distinct differences in range of motion, center-of-rotation behavior, ligament tension, and facet joint loading. These design-dependent effects complicate cross-study comparisons and limit the generalizability of conclusions regarding motion-preserving implants as a unified class. Additional barriers, including limited long-term outcome data beyond 10–15 years, computational challenges associated with modeling viscoelastic and articulating implants, and regulatory constraints, continue to hinder definitive comparison between fusion and motion-preserving strategies. While advanced finite element and AI-assisted modeling approaches offer improved efficiency and scalability, inconsistent validation across devices and loading conditions remains a key limitation.

Looking forward, the convergence of advanced imaging, finite element analysis, artificial intelligence, and additive manufacturing presents a transformative opportunity to move beyond standardized, one-size-fits-all implants. Specifically, biomechanics-driven, patient-specific spinal implants offer the potential to optimize stability, motion preservation, and load distribution based on individual anatomy and functional demands. Automated pipelines integrating CT or MRI segmentation, validated computational simulation, and implant design optimization have already demonstrated feasibility within clinically relevant timelines.

To realize this vision, it will require sustained interdisciplinary collaboration among engineers, spine surgeons, materials scientists, data scientists, and regulatory stakeholders to translate biomechanical insights into safe, effective, and widely accessible clinical solutions. Experimental and computational biomechanics must inform implant design and optimization, while clinicians provide imperative feedback on surgical feasibility and long-term patient implant outcomes. Regulatory frameworks must evolve in parallel to accommodate personalized and adaptive implant technologies without compromising safety or efficacy.

In conclusion, biomechanical innovation is redefining the landscape of spinal implant design and by integrating rigorous biomechanics, advanced computational modeling, and interdisciplinary clinical translation, the field is poised to shift from motion elimination toward motion optimization. Such a patient-centered, biomechanics-driven approach holds the greatest promise for reducing ASD, improving long-term outcomes, and advancing the next generation of spinal implant technologies.

## Figures and Tables

**Table 1:** Qualitative comparative biomechanical effects of cervical motion-preserving techniques and fusion constructs on index level and adjacent-level spinal kinematics and loading. Reported outcomes include changes in segmental range of motion, facet joint forces, and intradiscal pressure derived from cadaveric, finite element, and experimental studies.

Study	Metric	Arthroplasty/Motion-Preserving Technique	Fusion/Rigid Fixation Technique
Abudouaini et al., 2023 [30]	Index Level Flexion ROM	Maintained (near intact)	Decreased
	Index Level Extension ROM	Maintained (near intact)	Decreased
	Index Level Lateral Bending ROMN	Maintained (near intact)	Decreased
	Index Level Axial Rotation ROM	Maintained (near intact)	Decreased
	Superior Adjacent Spinal. Level Flexion ROM	Maintained (no significant change in ROM)	Maintained (no significant change)
	Inferior Adjacent Spinal Level Flexion ROM	Maintained (no significant change in ROM)	Maintained (no significant change)
	Superior Adjacent Facet Joint Pressure	Slightly increased	Increased
	Inferior Adjacent Facet Joint Pressure	Slightly increased	Increased
	Superior Adjacent Intradiscal Pressure	Slightly increased	Increased
	Inferior Adjacent Intradiscal Pressure	Slightly increased	Increased
Choi et al., 2021 [28]	Index Level Flexion ROM	Increased	Decreased
	Index Level Extension ROM	Increased	Decreased
	Index Level Facet Forces	Increased	Decreased
	Superior Adjacent Spinal Level Flexion ROM	Maintained or Slightly Decreased (Implant-dependent)	Increased
	Inferior Adjacent Spinal Level Flexion ROM	Maintained or Slightly Decreased (Implant-dependent)	Increased
	Superior Adjacent Spinal Level Extension ROM	Maintained or Slightly Decreased (Implant-dependent)	Increased
	Inferior Adjacent Spinal Level Extension ROM	Maintained or Slightly Decreased (Implant-dependent)	Increased
	Superior Adjacent Facet Forces	Variable (Implant-dependent)	Increased
	Inferior Adjacent Facet Forces	Variable (Implant-dependent)	Increased
	Superior Adjacent Intradiscal Pressure	Increased or Decreased (Implant-Dependent)	Increased
	Inferior Adjacent Intradiscal Pressure	Increased or Decreased (Implant-Dependent)	Increased
Gandhi et al., 2015 [7]	Index Level Flexion ROM	Increased	Decreased
	Index Level Extension ROM	Increased	Decreased
	Index Level Lateral Bending ROM	Increased	Decreased
	Index Level Axial Rotation ROM	Increased	Decreased
	Superior Adjacent Spinal Level Flexion ROM	Maintained (near intact)	Increased
	Inferior Adjacent Spinal Level Flexion ROM	Maintained (near intact)	Increased
	Superior Adjacent Spinal Level Extension ROM	Maintained (near intact)	Increased
	Inferior Adjacent Spinal Level Extension ROM	Maintained (near intact)	Increased
	Superior Adjacent Spinal Level Lateral Bending ROM	Maintained (near intact)	Increased
	Inferior Adjacent Spinal Level Lateral Bending ROM	Maintained (near intact)	Increased
	Superior Adjacent Spinal Level Axial Rotation ROM	Maintained (near intact)	Increased
	Inferior Adjacent Spinal Level Axial Rotation ROM	Maintained (near intact)	Increased
Gandhi et al., 2019 [31]	Index Level Flexion ROM	Increased	Decreased
	Index Level Extension ROM	Increased	Decreased
	Index Level Lateral Bending ROM	Increased	Decreased
	Index Level Axial Rotation ROM	Increased	Decreased
	Superior Adjacent Spinal Level ROM	Maintained or Decreased	Increased
	Inferior Adjacent Spinal Level ROM	Maintained or Decreased	Increased
	Global Motion Redistribution	Concentrated at Arthroplasty Level	Shifted to Adjacent Levels

**Table 2:** Design-dependent constraint mechanisms across CDA systems.

Factors Being Compared	Bryan Disc	Prestige LP Disc	ProDisc-C	Mobi-C Disc
Design Characteristics	Unconstrained Viscoelastic polyurethane nucleus Flexible endplates	Semi-constrained Metal-on-metal Rigid endplates	Semi-constrained Ball-in-socket Rigid core with central keel	Semi-constrained Mobile polyethylene core
Motion Mechanism	Elastic deformation of viscoelastic core and surrounding sheath	Controlled rotation through ball-in-trough metal articulation	Rotation through fixed ball-in-socket articulation	Controlled rotation through translation of mobile core
Pros	Lower index-level segmental stiffness More physiological load sharing Joint loading patterns closest to intact spine	Higher stability and control Lower stress and strain at facet and uncovertebral joints Decreased adjacent-level ROM and facet force	Preserves disc height and lordosis Adjacent IDP closer to intact spine Decreased adjacent-level ROM and facet force	Preserves index-level ROM and IDP Better compensation for intervertebral pressure of adjacent segment
Cons	Higher stress and strain energy density at facet and uncovertebral joints Higher stress at bone-implant interface	Higher index-level segmental stiffness Supraphysiologic index-level ROM and facet force Increased adjacent segment IDP Higher contact stress at bone-endplate interface	Higher index-level segmental stiffness Supraphysiologic index-level ROM and facet force Higher stress around central keel at bone-implant interface	Higher facet joint pressure at replacement level Lower intrinsic stability
Trade-offs and considerations	Lower stiffness improves physiological motion and load sharing, but reduces segmental stability	Higher stiffness improves control and stability, but alters physiological load sharing	Higher stiffness improves control and stability, but alters physiological load sharing	Intermediate stiffness results in intermediate stability and load sharing compared to fully rigid and low-stiffness designs

**Table 3:** Material-level biomechanical properties of cervical disc prostheses and their mechanistic implications for load transfer and subsidence risk, adapted from Dahl et al [14].

Study	Metric	Arthroplasty	Fusion
Dahl et al., 2011 [14]	Implant Material Dynamic Stiffness	Decreased (PU < PE, Ti)	Increased
	Implant Material Energy Absorption	Increased	Decreased
	Implant Material Viscous Damping	Increased	Decreased
	Axial Shock Absorption	Near intact (PU-based)	Decreased
	Load Transmission to Endplates	Reduced	Increased
	Subsidence Risk (Mechanistic)	Reduced	Increased

## Data Availability

No new data was curated.

## Abbreviations

The following abbreviations are used in this manuscript:

PU	Polyurethane
PE	Polyethylene
Ti	Titanium
FEA	Finite Element Analysis
